# Cortisol total/CRP ratio for the prediction of hospital-acquired pneumonia and initiation of corticosteroid therapy in traumatic brain-injured patients

**DOI:** 10.1186/s13054-020-2805-y

**Published:** 2020-03-04

**Authors:** Linpei Jia, Hongliang Zhang

**Affiliations:** 1 Department of Nephrology, Xuanwu Hospital, Capital Medical University, Changchun Street 45#, Beijing, 100053 China; 2 Department of Life Sciences, The National Natural Science Foundation of China, Shuangqing Road 83#, Beijing, 100085 China

Bouras and colleagues attempted to use the cortisol total/CRP ratio for the prediction of hospital-acquired pneumonia (HAP) and initiation of corticosteroid therapy in patients with severe traumatic brain injury (TBI) and with adrenal insufficiency [1]. They found that a cortisol total/CRP ratio > 3 upon admission may predict the development of HAP in patients with severe TBI [1]. The study is interesting, while we have some concerns about the study design and interpretation of the data.

The CORTI-TC trial [2] revealed that low-dose hydrocortisone with fludrocortisone did not improve the outcome of patients with TBI. Although the relatively small sample size is considered as a reason for negative findings, we believe that stratified analyses are more important for such a study. HAP is a form of nosocomial pneumonia, which is distinct from VAP [3]. In a published guideline from the Infectious Diseases Society of America and the American Thoracic Society, the term HAP denotes an episode of pneumonia not associated with mechanical ventilation [4, 5]. As acknowledged also by the authors, mechanical ventilation promotes a specific histological pattern of pneumonia [1]. Hence, patients with HAP and ventilator-associated pneumonia (VAP) should be categorized into two distinct groups. All cases of recorded HAP were VAP [1], which means all these patients received mechanical ventilation. Thus, we are eager to know about the patients without HAP. What is the percentage of mechanical ventilation use in these patients?

In this post hoc analysis of the CORTI-TC trial, the authors may hypothesize that the cortisol total/CRP ratio may or may not predict HAP. However, HAP is more associated with disease severity per se, e.g., consciousness state, prophylactic use of antibiotics, duration of intensive care, and mechanical ventilation, with many comorbidities potentially acting as confounding factors. We are then interested to know about the prophylactic use of antibiotics in the CORTI-TC trial.

Seemingly, results from Table 1 and Table 2 are conflicting [1]. Whereas in Table 1, up to 20% patients without HAP (versus 5.6% in those without HAP) had body temperature > 39.0 °C upon admission, in Table 2, multivariate analysis revealed that body temperature > 39.0 °C was closely associated with HAP [1]. Besides, the unit of the leucocyte count in Table 1 is grams per liter, which should be typographical errors.

In summary, HAP is common after TBI and closely associated with its outcome. More studies are warranted to seek surrogate biomarkers to predict the occurrence of HAP.

## Data Availability

Not applicable.
